# P-1440. Low measles seroprotection among adults with perinatally acquired HIV

**DOI:** 10.1093/ofid/ofaf695.1627

**Published:** 2026-01-11

**Authors:** Camille Knable, Gregory H Taylor, Patrick Ryscavage

**Affiliations:** University of Maryland Medical Center, Baltimore, MD; University of Maryland, Baltimore, Maryland; University of Maryland, Baltimore, Baltimore, MD

## Abstract

**Background:**

Due to decreasing vaccination rates in the US, there is widespread concern for the reestablishment of measles endemicity. Those with immune suppression, including people living with HIV (PLHIV), would be particularly vulnerable to poor measles-related outcomes. Among young adults living with HIV, rates of measles vaccine-related seroprotection are poorly understood. Critically, adults with perinatally-acquired HIV (PHIV) may be at increased risk of poor measles vaccine seroresponse. We sought to examine patterns of measles vaccination as well as measles vaccine seroresponse among a cohort of young adult PHIV and age-range matched young adults with non-perinatally acquired HIV (NPHIV).

**Table 1:**
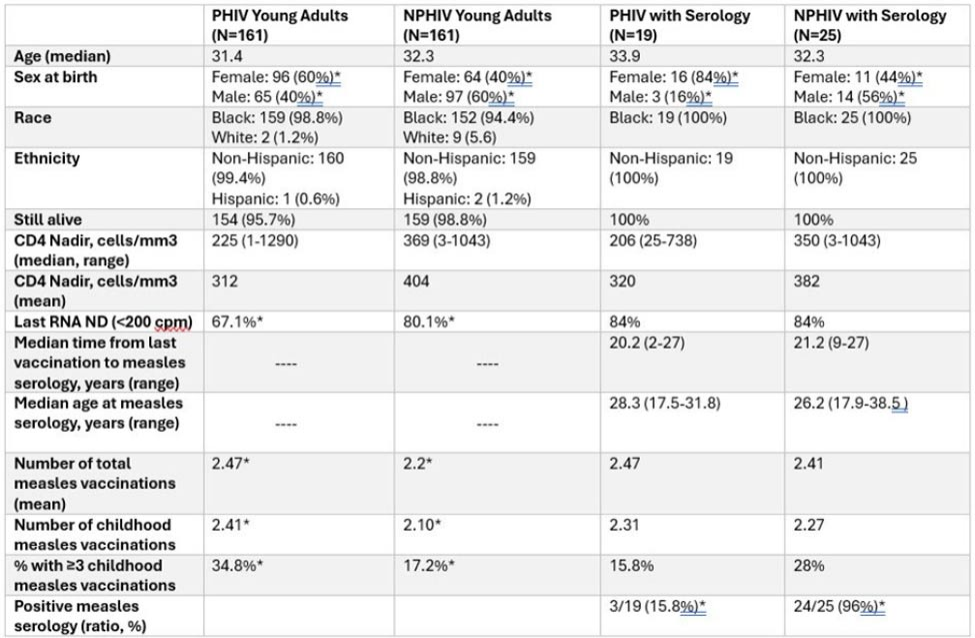
Cohort demographic and clinical characteristics *p <.05; PHIV: perinatally acquired HIV; NPHIV: non-perinatally acquired HIV; cpm: copies per mL

**Methods:**

We conducted a retrospective cohort study which included young adult PHIV and age-range matched NPHIV. The primary outcome was measles seropositivity. Rubeola serology was reported as quantitative IgG or qualitative ELISA-based results. Inclusion criteria included documentation of measles, mumps, and rubella (MMR) vaccination and/or rubeola serology. We collected demographics, HIV clinical data, MMR vaccination dates, and rubeola serologies. Fischer’s exact test and Mann Whitney were used to assess the association between categorical variables and continuous variables, respectively.

**Results:**

Three hundred and twenty-two patients were included in the study (161 PHIV and 161 NPHIV). Demographic and clinical data are shown in Table 1. Median age at measles serology was 28.3 years among PHIV and 26.2 years among NPHIV. PHIV had a significantly higher proportion of females and were less likely to have HIV RNA suppression at last check. Nadir CD4 was non-significantly lower among PHIV. PHIV had a higher mean number of childhood MMR vaccinations and were more likely to have received ≥3 childhood MMR vaccinations (35% vs 17%). Measles vaccination seroresponse was significantly lower among PHIV than among NPHIV (15.8% vs 96%, p=.0001).

**Conclusion:**

Adults with perinatally acquired HIV had a low prevalence of measles seroprotection despite evidence of at least 2 childhood MMR vaccinations. In the context of a growing threat of measles endemicity in the US, HIV providers should assess rubeola serology among adult PHIV and consider revaccination when clinically appropriate.

**Disclosures:**

Patrick Ryscavage, MD, Gilead Sciences, Inc.: Grant/Research Support

